# Comment on Salazar et al. Weight Regain after Metabolic Surgery: Beyond the Surgical Failure. *J. Clin. Med.* 2024, *13*, 1143

**DOI:** 10.3390/jcm13123608

**Published:** 2024-06-20

**Authors:** Athanasios G. Pantelis

**Affiliations:** Athens Medical Group, Obesity and Metabolic Disorders Department, Psychiko Clinic, 1 Andersen Str., Psychiko, 11525 Athens, Greece; ath.pantelis@gmail.com

I read the article by Salazar J. et al. with great interest and would like to congratulate the authors for their detailed and comprehensive review [1]. At the same time, I wish to highlight some issues regarding certain definitions that, beyond typology, reflect current debates and concepts on obesity as a disease and its management in the era of novel treatment modalities.

The title of the article contains two of those definitions, i.e., “weight regain” and “surgical failure”. Both terms should be examined under the spectrum that obesity is a disease rather than a state, choice, or behavioral abnormality [2]. Obesity has been recognized as a chronic relapsing progressive disease by the World Health Organization [3], the World Obesity Federation [4], the American Health Association [5], and other prominent professional bodies. Consequently, the proposed term, as it has been stressed particularly by the American Society of Metabolic Bariatric Surgery (ASMBS), is “weight recurrence” instead of “weight regain”, because it reflects the nature of obesity as a disease rather than a state [6]. Along the same lines, the term surgical or treatment “failure” should be avoided because it undermines the evolutionary and homeostatic stressors of obesity (the so-called obesity set-point theory) [7], weakens the status of obesity as a disease, and reinforces weight stigma and discrimination [8]. Most importantly, it has been long recognized that insufficient weight loss (IWL) or weight recurrence (WR) following metabolic bariatric surgery (MBS) are not necessarily connected to failed metabolic effects, such as diabetes remission or normalization of blood pressure, lipid profile, and sleep apnea [9]. As such, the term “surgical failure” degrades the pivotal role of MBS on restoring health and well-being.

Further in the manuscript, the authors reference a meta-analysis dating from 2014 to support an overall complication rate after MBS that reaches 17%. This conceals two caveats: On the one hand, the collective experience with MBS was far lower until then, given the exponential increase in the number of metabolic bariatric operations from 2016 onwards [10,11]. On the other hand, older techniques (like the adjustable gastric band) tend to be abandoned in modern practice, whereas newer techniques (such as one-anastomosis gastric bypass (OAGB)) have been endorsed by both ASMBS and IFSO (International Federation for the Surgery of Obesity and Metabolic Disorders), which are the two official, international, and closely cooperating organizations for MBS [12,13]. In reality, irrespective of the specific operation, the overall complication rate after MBS is 2–3%, whereas Clavien–Dindo class 3 and 4 complications as well as mortality are way below 1% [14,15,16].

The next point of interest is the definition of WR. In the literature, there is an abundance of definitions and reviews of definitions regarding WR, a detailed analysis of which is beyond the scope of the commentary in hand (for an in-depth insight, please refer to our previous publication [17]). In brief, Nedelcu et al., in their seminal paper dating back to 2016, retrieved five definitions of WR, ranging from any increase in weight to proportional and percentile increases in the body mass index (BMI) or excess weight loss (EWL), respectively [18]. More recently, a systematic review conducted by the POWER Task Force of the ASMBS retrieved 29 different definitions for WR and 3 for IWL, whereas the terms “primary” and “secondary non-responders” were officially introduced in pertinent nomenclature, further stressing the complexity of the problem [6]. Beyond being a matter of terminology, each definition bears variable sensitivity and specificity, whereas implementing different definitions yields a wide range of results regarding the prevalence of the WR term [19]. This is the reason why other research groups have implemented more refined, algorithmic approaches to define suboptimal bariatric outcomes following MBS and have shifted towards a prompt recognition and personalized treatment of WR [20,21]. In any case, the officially adopted definitions by IFSO are (i) weight or BMI loss of <20% regarding the suboptimal initial clinical response and (ii) recurrent weight gain of >30% in terms of late post-operative clinical deterioration, as declared in a recent Delphi-based consensus statement [22].

The bottom line is that weight recurrence constitutes an imminent challenge in the era of an increasing prevalence in both obesity and revisional bariatric surgery. According to seminal studies with long-term follow-up surveillance, the prevalence of WR might range from approximately 4% (after Roux-en-Y gastric bypass) to almost 28% (after sleeve gastrectomy) [23,24]. However, this is a rough estimation, given the wide range of results in the documented prevalence of WR, the lack of consensus regarding the definitions of WR, and the deviations in the technique used that may lead to significant differentiations in the bariatric outcome (i.e., narrow versus wide sleeve, various limb lengths in bypass procedures, etc.). In this regard, the need to adopt uniform nomenclature and obtain an objective assessment of the magnitude of this problem is imperative, particularly in view of the onset of new anti-obesity medications that are predicted to assume a game-changing role on weight recurrence following metabolic bariatric surgery.

Lastly, I would like to address the registered mechanisms underlying WR. The authors have performed a meticulous and comprehensive recitation of the anatomical, neurohormonal, behavioral, and genetic factors that may contribute to WR. In addition to those, we would like to mention the potential implications of the weight set-point and obesity phenotypes [7,25,26]. Although the weight set-point remains a theory with a strong evolutionary component that needs to be validated in large-scale population studies, it might hold a central place in determining WR as it has been anecdotally documented in clinical practice by most bariatric surgeons and obesity specialists. Moreover, while MBS seems to be beneficial for both metabolically healthy and unhealthy phenotypes of obesity (i.e., the dynamics of obesity phenotyping) [27], the impact of these phenotypes on WR post-MBS (i.e., the kinetics of obesity phenotyping) remains elusive. The impact of both the weight set-point and obesity phenotyping warrant a further investigation in future studies aiming to demystify the pathophysiology of WR.
